# Diabetes Treatment Satisfaction Is Associated With Health-Related Quality of Life Among Parents and Children With Type 1 Diabetes

**DOI:** 10.1155/pedi/7760362

**Published:** 2025-11-14

**Authors:** Helena Agenäs, Maria Ödling, Anna Lena Brorsson, Inger Kull, Anna Lindholm-Olinder

**Affiliations:** ^1^Department of Clinical Science and Education, Södersjukhuset, Karolinska Institutet, Stockholm, Sweden; ^2^Astrid Lindgren Children's Hospital, Karolinska University Hospital, Stockholm, Sweden; ^3^Department of Women's and Children's Health, Karolinska Institutet, Stockholm, Sweden; ^4^Sachs' Children and Youth Hospital, Stockholm, Sweden

**Keywords:** children, health-related quality of life, parent, socioeconomic factors, treatment satisfaction, Type 1 diabetes

## Abstract

**Background:**

Type 1 diabetes (T1D) requires numerous daily self-management decisions and, for children, parents have a crucial role in this. The aim of this study was to evaluate treatment satisfaction among parents of children with T1D and the associations with the child's health-related quality of life (HRQoL) and perceived diabetes control. A secondary aim was to evaluate treatment satisfaction among school children with T1D and the associations with HRQoL and perceived diabetes control.

**Methods:**

In this cross-sectional study, 111 parents of children with T1D (0–12 years) and 75 children with T1D (8–12 years) were included. Treatment satisfaction was measured using the diabetes treatment satisfaction questionnaire (DTSQ), parent version, scored 0–60, or teens version, scored 0–48. HRQoL was measured with DISABKIDS. Differences between groups were analyzed using independent *t*-tests and one-way analysis of variance. Linear regression was used to investigate associations between treatment satisfaction and HRQoL and perceived diabetes control.

**Results:**

The mean value for treatment satisfaction among parents was 41.0 (95% confidence interval (CI): 39.5–42.5) and among children 36.3 (95% CI: 34.5–38.0). Parents' treatment satisfaction was associated with their child's HRQoL (by proxy), perceived diabetes control, and diabetes duration (correlations coefficient [*R*^2^] = 0.54, *p*  < 0.001). Children's treatment satisfaction was associated with HRQoL (*R*^2^ = 0.29, *p*=0.005).

**Conclusion:**

The child's HRQoL and perceived diabetes control are important for treatment satisfaction among parents of children with T1D.

## 1. Background

In Sweden, nearly 8500 children and adolescents have Type 1 diabetes (T1D) [1]. A diagnosis of T1D means a lifetime of administering insulin, monitoring glucose levels, calculating food intakes, and facing the risk of physical complications [2].

Parents who have a child with T1D have a substantial responsibility in managing their child's disease. The continuous diabetes-related tasks, as well as the fear of incorrect treatment and too high or too low glucose levels can lead to distress and reduced health-related quality of life (HRQoL) in parents [3–5]. Factors such as stress levels and the quality of parent–child interactions have been reported to be associated with treatment satisfaction [3, 6].

In recent years, there has been a rapid development of technical equipment for insulin dosing and glucose measurement [7]. Research indicates that treatment satisfaction among children and adolescents is linked to the treatment method [8]. Previous studies indicate that treatment with continuous subcutaneous insulin infusion (CSII) may improve glycaemic control, treatment satisfaction, and HRQoL in children with T1D and their parents [8–10]. Similarly, the use of continuous glucose monitoring (CGM) has been shown to enhance glycaemic outcomes and contribute to improved QoL [11]. Today, the majority of Swedish children, 80%, are treated with CSII and 98% use CGM for glucose control measurement [1]. Although access to advanced technology has increased, the fear of hypoglycemia often persists, continuing to impact treatment satisfaction [12].

Factors like living with both parents, having parents with a high socioeconomic status, and equitable sharing of diabetes management responsibilities between parents have been shown to positively affect children's HRQoL and glycated hemoglobin (HbA1c) levels [13]. When parents receive support from health care professionals in managing their child's diabetes, this often improves glycaemic control in the child, which enhances parental treatment satisfaction [7].

However, to our knowledge, no studies have investigated treatment satisfaction among parents of children with T1D in relation to HRQoL by proxy or perceived diabetes control. From a clinical perspective, it is of importance to better understand the parental perspective, to be able to provide resources where needed.

The aim of this study was to evaluate treatment satisfaction among parents of children with T1D and the associations with the child's HRQoL and perceived diabetes control. A secondary aim was to evaluate treatment satisfaction among schoolchildren with T1D and the associations with HRQoL and perceived diabetes control.

## 2. Methods

This cross-sectional study was performed at Sachs' Children and Youth Hospital, Södersjukhuset, Stockholm, Sweden.

In connection with visits to the outpatient clinic, participants (parents of children with T1D aged 0–12 years and children with T1D aged 8–12 years) received written information about the study and signed informed consent documents. A total of 197 parents of children with T1D were invited to participate in the study, as well as 134 children with T1D. If the questionnaire was not completed correctly, it was not used in the study. The children were encouraged to complete the questionnaire independently, but received help from their parents with reading the questions if needed. Only one parent per child completed the questionnaire and provided proxy-reported HRQoL for their child. The parents and children were given the option to complete the questionnaires either during their clinic visit or at home and return them by post. Data were collected between December 2018 and July 2020. Within the time period, a reminder was sent by post to those who had not responded after 2–3 weeks.

### 2.1. Measurement

#### 2.1.1. Parents' Treatment Satisfaction

The diabetes treatment satisfaction questionnaire status (DTSQs-Parent), a questionnaire developed in England [14], was used to measure parents' perceived treatment satisfaction. DTSQs-Parent has been translated and validated in a Swedish context [15] and was recently psychometrically evaluated [16].

The DTSQs-Parent version, with 14 items, is divided into four dimensions (treatment satisfaction, perceived diabetes control, perceived frequency of low blood sugar levels, and concerns about the treatment's effects on the parent's life). Participants respond on a seven-point Likert scale ranging from 0 to 6. After inversion of scores for two items, higher scores indicate greater agreement in all dimension [16]. Treatment satisfaction is assessed using 10 items from the DTSQs-Parent (items 1, 5–9, and 11–14; score 0–60), which cover aspects such as satisfaction, ease, flexibility, school day, liked activities, family life, understanding, discomfort, medical support, and willingness to continue treatment. Perceived diabetes control is assessed with two items (perceived control and perceived frequency of high blood glucose levels), with a score range of 0–12. Two items, perceived frequency of low blood sugar levels and concerns about the effects on the parent's own life, are calculated separately [16]. Licens to use DTSQ-Parent was obtained from Health Psychology Research Ltd (Ref. CB 367).

### 2.2. Children's Treatment Satisfaction

The child version of DTSQs-Teen, originally developed in England [14], was used to measure how children (aged 8–12 years) perceived treatment, diabetes control, and the level of pain and discomfort in daily life. DTSQs-Teen has been translated into Swedish [15].

DTSQs-Teen version, with 12 items, assesses treatment satisfaction and perceived glucose control among adolescents [15]. Participants respond on a seven-point Likert scale ranging from 0 to 6. After inversion of scores for two items, higher scores indicate greater agreement. Treatment satisfaction is assessed using eight items (items 1, 5–9, and 11–12, score 0–48) covering aspects such as satisfaction, ease, flexibility, school day, liked activities, understanding, medical support, and willingness to continue treatment [15]. Two items (perceived control and perceived frequency of high blood glucose levels) are calculated to assess perceived glucose control, with a score range of 0–12. Two items, perceived frequency of low blood sugar levels and the level of pain and discomfort, are calculated separately [15].

Licens to use DTSQ-Teen was obtained from Health Psychology Research Ltd (Ref. CB 367).

### 2.3. HRQoL

HRQoL was measured using the short generic questionnaire DISABKIDS for children with chronic conditions, along with a proxy version for parents. The form comprises 12 five-point Likert scale items, ranging from 1 (never) to 5 (always), assigned to three domains: mental, social and physical. The scores were transformed to a 0–100 scale, based on an equation provided in the creators' instructions, with higher scores indicating higher HRQoL [17].

### 2.4. Background and Clinical Factors

Socioeconomic factors, HbA1c, duration, sex, and age were considered as confounders.

The parents provided information on socioeconomic factors (economic situation, education level, and family situation). Parents' self-perceived economic situations was sorted into three categories: better than average, average, or worse than average. Education level was sorted into two subgroups: lower than university or higher education. Family situation was categorized as parents living together, shared custody or single. Parents also provided information on their child's sex, age, diabetes duration, type of treatment, and current glycaemic control measured as HbA1c levels. HbA1c levels (normal values: 27–42 mmol/mol) were measured from capillary blood samples and analyzed with the DCA Vantage apparatus (Siemens Healthcare AB). In Sweden, the goal for HbA1c level in children with T1D is ≤48 mmol/mol [18].

### 2.5. Data Analysis

Descriptive statistics were used to present data as numbers and percentages for all dimensions of DTSQs-Parent and DTSQs-Teen. Differences between groups were analyzed using independent *t*-tests and one-way analysis of variance. Multiple linear regression was used to investigate associations between treatment satisfaction and HRQoL and perceived diabetes control, based on a previous Swedish study in teenagers (15–20 years), which showed associations between treatment satisfaction and HRQoL and glycaemic control [15]. Initially, the regression model included socioeconomic factors and perceived diabetes control, along with age, sex, HRQoL, diabetes duration, and HbA1c. However, socioeconomic factors were excluded as they did not significantly contribute to the model, and perceived diabetes control was significant only in parental reports, not in children's responses. Therefore, separate analyses were conducted, with the final model for children adjusted for age, sex, HRQoL, diabetes duration, and HbA1c.

For the descriptive statistics, the child's age was dichotomized as 0–6 or 7–12 years, and diabetes duration as 0.5–3 or 4–12 years. The child's HbA1c was dichotomized as ≤48 or >48 mmol/mol. HRQoL was dichotomized as <70 and ≥70. Results were expressed as mean values with 95% confidence intervals (CIs). *p*-Values of less than 0.05 were considered statistically significant. Data analysis was conducted using the statistical package SPSS (version 28).

## 3. Results

The study population consisted of 111 parents (66% mothers), of children (0–12 years, mean age 8.6 years) with T1D, and 75 children (8–12 years, mean age 10.3 years) with T1D (Table 1). The children's mean diabetes duration was 3.4 years, their mean HbA1c level was 48.9 mmol/mol, and the majority were using CSII (*n* = 102, 92%) and CGM (*n* = 109, 98%) at the time of recruitment to the study. In the subgroup of children who answered questionnaires (*n* = 75), the mean diabetes duration was 4.0 years, their mean HbA1c level was 50.5 mmol/mol, 67 (89%) used CSII and 73 (97%) used CGM.

### 3.1. Parents

#### 3.1.1. Treatment Satisfaction

The mean value for treatment satisfaction was 41.0 (95% CI: 39.5–42.5; Table 2).

Parents' treatment satisfaction was lower if the child was a girl (mean 39.1 vs. 42.4, *p*=0.034), if they perceived that the child had impaired HRQoL (score <70; mean 37.4 vs. 43.8; *p*  < 0.001) or if they estimated their economic situation to be worse than average (mean 32.6 vs. 41.5; *p*=0.012). Mothers with lower education levels (lower than university) had lower treatment satisfaction than mothers with higher education (mean 38.2 vs. 42.4; *p*=0.009).

Multiple linear regression analyses showed that the parents' treatment satisfaction was associated with their perception of diabetes control, their perception of their child's HRQoL, and the child's diabetes duration (longer duration higher treatment satisfaction), adjusted for child sex, age, and HbA1c (correlation coefficient [*R*^2^] = 0.54; *p*  < 0.001; Table 3).

### 3.2. Perceived Diabetes Control

Perceived diabetes control was lower if the parents estimated that the child had impaired HRQoL (mean 5.2 vs. 6.6; *p*=0.001) or higher HbA1c levels (mean 5.1 vs. 6.8; *p*  < 0.001), or if they reported worse economic status (mean 4.0 vs. 6.4; *p*=0.030; Table 2). Additionally, mothers with lower education levels reported lower perceived diabetes control (mean 5.3 vs. 6.5, *p*=0.011; Table 2).

### 3.3. Hypoglycemia

Mothers with lower education levels more often perceived their child's blood glucose levels to be low (mean 4.1 vs. 4.7, *p*=0.010; Table 2).

### 3.4. The Impact of the Treatment

Parents who rated their child's HRQoL lower (<70) reported a greater impact from the treatment on their own life (mean 2.3 vs. 3.6; *p*  < 0.001; Table 2). Additionally, the treatment had a greater impact on the lives of mothers with lower education levels (mean 2.4 vs. 3.3; *p*=0.007; Table 2).

### 3.5. Children

#### 3.5.1. Treatment Satisfaction

The mean value for treatment satisfaction in children aged 8–12 years was 36.3 (95% CI: 34.5–38.0; Table 4). Children's treatment satisfaction was lower if they perceived their HRQoL to be lower (mean 32.9 vs. 38.4; *p*=0.003).

Children's treatment satisfaction was associated with HRQoL, adjusted for sex, age, HbA1c, and diabetes duration (*R*^2^ = 0.29; *p*=0.005; Table 5).

### 3.6. Perceived Diabetes Control

Perceived diabetes control (items 2 and 3) in children was lower if their HbA1c levels were higher (≥48 mmol/mol; mean 5.5 vs. 8.0; *p*  < 0.001; Table 4).

### 3.7. Hypoglycemia

Factors such as the child's sex, duration of diabetes, HbA1c, HRQoL or the parents' socioeconomic factors did not affect how often the child perceived their blood sugar to be low (item 4; Table 4).

### 3.8. Discomfort and Pain

Children with lower HRQoL (<70) experienced more discomfort or pain caused by the treatment (item 10) than children with higher HRQoL (>70; mean 3.3 vs. 4.4; *p*=0.009; Table 4).

## 4. Discussion

This cross-sectional study investigated treatment satisfaction in parents of children (0–12 years) with T1D and children (8–12 years) with T1D, as well as possible associations between treatment satisfaction and HRQoL and perceived diabetes control. Among parents, there were associations between treatment satisfaction and both the child's HRQoL and perceived diabetes control. However, in children (8–12 years) only an association between treatment satisfaction and HRQoL was found. Descriptive statistics showed that parents reported lower treatment satisfaction if their child was a girl, if they perceived the child's HRQoL to be low, if the mother had a lower education level (lower than university) or if the family had a lower economic status. For children, lower HRQoL was the only factor influencing treatment satisfaction.

In the present study, parents who perceived their child's HRQoL as low experienced decreased treatment satisfaction, poorer perceived diabetes control and felt a greater impact on their own life. Similar results have been found in a previous study where parents' perceptions of their child's HRQoL and burden of diabetes management were associated with higher reported disease severity [19].

There are sex differences among youths with T1D, as girls tend to have lower HRQoL and poorer glycaemic control than boys [20]. In our study, parents of girls reported lower treatment satisfaction compare to parents of boys. One possible explanation could be that girls experience and express more emotional distress or treatment burden, which may influence parental perceptions. Alternatively, parents may perceive girls as more vulnerable or less satisfied with treatment outcomes, thereby indirectly affecting their own satisfaction.

Additionally, in the current study, high HbA1c levels in a child were associated with both the parents' and the child's perceived diabetes control, which could cause stress. A population-based study found that increased parental burden and emotional distress were associated with higher HbA1c levels, related to the child's diabetes management [21]. Other studies have shown that lower HRQoL is associated with higher HbA1c [4, 13, 18]. These findings highlight the importance of addressing both the physical and the psychological aspects of diabetes management to support both children and their parents. Providing emotional support may help reduce the burden associated with managing high HbA1c levels [21].

Mothers with lower education levels had impaired treatment satisfaction, lower perceived diabetes control, and experienced greater impact on their own life. Previous studies have also shown that lower than university level education in a mother is strongly associated with poorer glucose control in their child and lower reported parental HRQoL [22, 23]. Our study also found that mothers with lower education levels more often perceived their children to have more frequent hypoglycemic episodes than mothers with higher education levels. These findings are consistent with research showing that parental fear of hypoglycemia is associated with socioeconomic factors [5].

Previous research has identified an association between reduced HRQoL in children with T1D and socioeconomic factors such as living in a single-parent households or in a family facing economic disadvantages [24]. In our study, we could not see any differences in children's levels of treatment satisfaction related to socioeconomic factors. However, among parents, low socioeconomic status was associated with lower treatment satisfaction, despite the majority using advanced technology, CSII, and CGM. On the other hand, our study showed that children with diabetes who reported lower HRQoL tended to have lower treatment satisfaction.

These findings highlight the importance of providing support to parents, particularly mothers, with lower education levels and a worse economic situation. A multidisciplinary diabetes team play a crucial role in supporting both children with T1D and their parents [7]. With the assistance of a diabetes team, families can receive education to maximize the effectiveness of diabetes care and stay updated on advances in diabetes management and technology [7]. Therefore, it is crucial to measure treatment satisfaction in both the child and their parents, in addition to monitoring glucose control, to provide personalized support [25].

### 4.1. Strengths and limitations

One strength of the current study is that we were able to assess both parents' and children's treatment satisfaction and how each associated with HRQoL in a clinical setting. It is more common to evaluate treatment satisfaction in both children and parents in studies exploring the effects of new technology, such as CSII, CGM, and sensor-augmented pump therapy [11, 26].

The generalizability to other parts of the world with other reimbursement policies can be questioned. In Sweden, advanced treatment with CSII and CGM are free of charge and most participants in the study used advanced treatment. However, despite this, treatment satisfaction still varied, suggesting that treatment satisfaction is not affected by the type of treatment but rather by its outcome. A limitation of the study could be that most responses were from mothers (66%). However, we did not find any differences in treatment satisfaction and perceived diabetes control between mothers and fathers, may be due to low power. Another limitation could be that the participants were recruited from the same clinic, in a metropolitan area in Sweden. The DTSQ-Teen has not been psychometrically validated in a Swedish context and is not validated for children aged 8–12 years; therefore, the results should be interpreted with caution. Further, too few participants completed the DTSQs-Teen to enable identification of potential associations between treatment satisfaction and perceived diabetes control. It may also be a limitation that we did not use the actual hypoglycemia scores, but these do not necessarily reflect perceived hypoglycemia. We chose to assume that perceived hypoglycemia was more relevant to treatment satisfaction.

Data collection was conducted both before and during the COVID-19 pandemic. However, we believe the pandemic had minimal impact on our results, as Sweden's approach to managing it did not include a full societal lockdown. This meant that children with T1D continued to have regular check-ups.

## 5. Conclusion

Parental treatment satisfaction was significantly associated with their perceptions of their child's HRQoL, perceived diabetes control, and diabetes duration. Children's treatment satisfaction was associated with their own HRQoL and HbA1c levels. Mothers with lower education levels had impaired treatment satisfaction, lower perceived diabetes control, and experienced greater impact on their own life, which highlight the importance of providing support to these parents. These findings may underscore the importance of assessing treatment satisfaction among parents and children and the child's HRQoL both from the parents' and the child's own view. Evaluating these factors may help to better understand experiences, support person-centered care, and foster a sense of security around diabetes self-management.

## Figures and Tables

**Table 1:** Background and clinical factors of parents and their children (aged 0–12 years) with Type 1 diabetes (*n* = 111) and participating children between 8 and 12 years (*n* = 75) at recruitment.

Characteristic	Questionnaire	Questionnaire
	Parents to children 2–12 years	Children between 8 and 12 years
Parents: mother, *n* (%)	73 (65.8)	—
Child's sex: girl, *n* (%)	46 (41.4)	34 (45.3)
Age of the child, mean (range)	8.6 (2–12)	10.3 (8–12)
Duration of the child's diabetes (year), mean (range)	3.4 (0.5–12)	4.0 (0.5–12)
Child's HbA1c, mean (range)	48.9 (30–114)	50.5 (32–114)
Children treated with CSII, *n* (%)	102 (91.9)	67 (89.3)
Child's glucose measurement, *n* (%)		
rtCGM	94 (84.7)	63 (84.0)
isCGM	15 (13.5)	10 (13.3)
SMBG	2 (1.8)	2 (2.7)
Family situation, *n* (%)	*n* = 108	*n* = 73
Parents living together	86 (79.6)	54 (74.0)
Shared custody^a^	16 (14.8)	12 (16.4)
Single parents	7 (6.5)	7 (9.6)
Perceives economic situation, *n* (%)	*n* = 108	*n* = 73
Better than average	50 (46.3)	33 (45.2)
Average	52 (48.1)	38 (52.0)
Worse than average	7 (6.5)	2 (2.7)
Level of education: mother, *n* (%)	*n* = 105	*n* = 69
University	69 (65.7)	39 (56.5)
Lower than university	36 (34.3)	30 (43.5)
Level of education: father, *n* (%)	*n* = 103	*n* = 67
University	63 (61.2)	40 (59.7)
Lower than university	40 (38.8)	27 (40.3)

Abbreviations: CSII, continuous subcutaneous insulin infusion; isCGM, intermittently scanned continuous glucose monitoring; rtCGM, real-time contentious glucose monitoring; SMBG, self-monitoring of blood glucose.

^a^Living 1 week with mother and 1 week with father.

**Table 2:** Parents treatment satisfaction with the child's Type 1 diabetes (*n* = 111).

Background and clinical factors	DTSQ-Parent^a^, mean (95% CI)	Perceived diabetes control^b^, mean (95% CI)	Item 4^c^, mean (95% CI)	Item 10^d^, mean (95% CI)
Overall	41.0 (39.5, 42.5)	6.0 (5.6, 6.4)	4.5 (4.3, 4.7)	3.0 (2.7, 3.3)

Age group				
0–6 year	41.9 (38.7, 44.6)	6.0 (5.1, 6.8)	4.6 (4.1, 5.0)	3.2 (2.6, 3.8)
7–12 year	40.7 (38.9, 42.4)	6.0 (5.6, 6.6)	4.5 (4.3, 4.7)	3.0 (2.6, 3.3)
*p*-Value (*T*-test)	0.465	0.862	0.776	0.395

Child's sex				

Girls	39.1 (36.6, 41.5)	5.7 (5.0, 6.4)	4.4 (4.2, 4.7)	2.8 (2.3, 3.2)
Boys	42.4 (40.5, 44.2)	6.2 (5.7, 6.8)	4.5 (4.3, 4.8)	3.2 (2.8, 3.6)
*p*-Value (*T*-test)	**0.034**	0.209	0.605	0.203

Diabetes duration				

0–3 year	40.7 (38.7, 42.6)	6.0 (5.5, 6.5)	4.4 (4.1, 4.6)	3.1 (2.7, 3.5)
4–12 year	41.6 (39.3, 43.8)	6.1 (5.4, 6.8)	4.7 (4.4, 5.0)	2.9 (2.5, 3.4)
*p*-Value (*T*-test)	0.558	0.869	0.096	0.607

HbA1c (mmol/mol)				

<48	42.1 (40.4, 44.1)	6.8 (6.2, 7.3)	4.5 (4.2, 4.8)	3.0 (2.6, 3.4)
>48	39.7 (37.3, 41.8)	5.1 (4.6, 5.6)	4.5 (4.2, 4.7)	3.0 (2.6, 3.4)
*p*-Value (*T*-test)	0.104	**<0.001**	0.749	0.962

HRQoL by proxy (score)				

<70	37.4 (35.2, 39.5)	5.2 (4.7, 5.8)	4.4 (4.1, 4.7)	2.3 (1.9, 2.7)
>70	43.8 (41.9, 45.5)	6.6 (6.0, 7.2)	4.6 (4.3, 4.8)	3.6 (3.2, 3.9)
*p*-Value (*T*-test)	**<0.001**	**0.001**	0.433	**<0.001**

Education parents				

Mother lower	38.2 (35.4, 41.0)	5.3 (4.6, 6.0)	4.1 (3.8, 4.5)	2.4 (1.9, 3.0)
Mother higher	42.4 (40.7, 44.2)	6.5 (5.9, 7.0)	4.7 (4.4, 4.9)	3.3 (2.9, 3.6)
*p*-Value (*T*-test)	**0.009**	**0.011**	**0.010**	**0.007**
Father lower	39.5 (36.9, 42.0)	5.8 (5.1, 6.6)	4.4 (4.0, 4.7)	2.8 (2.2, 3.3)
Father higher	42.0 (40.1, 43.8)	6.2 (5.6, 6.7)	4.5 (4.3, 4.8)	3.1 (2.8, 3.5)
*p*-Value (*T*-test)	0.119	0.502	0.350	0.308

Economic group				

Better than average	41.5 (39.7, 43.3)	6.4 (5.8, 7.0)	4.6 (4.3, 4.9)	3.2 (2.8, 3.6)
Average	41.6 (39.2, 43.9)	6.0 (5.4, 6.6)	4.5 (4.2, 4.8)	3.0 (2.5, 3.4)
Worse than average	32.6 (24.2, 41.0)	4.0 (1.3, 6.7)	3.7 (2.3, 5.1)	1.7 (0.2, 3.2)
*p*-Value (ANOVA)	**0.012**	**0.030**	0.104	0.056

Family situation				

Parents living together	41.5 (40.0, 42.9)	6.0 (5.6, 6.5)	4.5 (4.3, 4.7)	3.0 (2.7, 3.4)
Shared custody/single	38.8 (34.8, 42.6)	6.0 (5.1, 6.8)	4.4 (4.0, 4.8)	2.9 (2.2, 3.7)
*p*-Value (*T*-test)	0.153	0.864	0.700	0.742

*Note:* One way ANOVA or *T*-test. Data in bold are statistically significant.

^a^DTSQ-Parent = Item 1, 5–9, 11−14.

^b^Perceived diabetes control Item 2, 3 (How well controlled do you feel your child's diabetes has been lately? How often have you felt that your child's blood sugars have been too high lately?).

^c^Item 4 = How often have you felt that your child's blood sugars have been too low lately?

^d^Item 10 = How satisfied are you with the way your child's treatment is affecting your own life?

**Table 3:** The association between treatment satisfaction and background and clinical factors among parents analyzed with linear regression.

Variable	*B*	*β*	95% CI	*p*-Value
Constant	10.19	—	0.29,20.10	0.044
Child's age	−0.10	−0.04	−0.52,0.32	0.649
Child's sex^a^	1.48	0.09	−085,3.80	0.210
HRQoL by proxy	0.24	0.47	0.17,0.32	**<0.001**
PDC parent	1.44	0.42	0.88,2.00	**<0.001**
Diabetes duration	0.57	0.21	0.09,1.06	**0.021**
HbA1c^b^	0.04	0.05	−0.08,015	0.542
*R* ^2^	0.54	—	—	**<0.001**

*Note:* Dependent variable: treatment satisfaction (Item: 1, 5–9, 11−14). Data in bold are statistically significant.

Abbreviations: CI, confidence interval; HRQoL, health-related quality of life; PDC, perceived diabetes control.

^a^Girls = 1, boys = 2.

^b^Glycemic control.

**Table 4:** Children's diabetes treatment satisfaction (*n* = 75).

Background and clinical factors	DTSQ-Teen^a^, mean (95% CI)	Perceived diabetes control^b^, mean (95% CI)	Item 4^c^, mean (95% CI)	Item 10^d^, mean (95% CI)
Overall	36.3 (34.5, 38.0)	6.8 (6.1, 7.6)	2.9 (2.5, 3.3)	3.9 (3.5, 4.3)

Child's sex				

Girls	34.9 (31.8, 38.0)	6.2 (5.0, 7.5)	2.9 (2.1, 3.6)	3.7 (3.1, 4.3)
Boys	37.3 (35.3, 39.4)	7.3 (6.4, 8.2)	2.9 (2.4, 3.4)	4.0 (3.4, 4.6)
*p*-Value	0.185	0.164	0.961	0.473

Diabetes duration				

0–3-year	36.1 (33.2, 38.9)	7.3 (6.3, 8.4)	2.5 (1.9, 3.1)	3.6 (3.0, 4.4)
4–12-year	36.4 (33.9, 38.9)	6.3 (5.3, 7.3)	3.1 (2.4, 3.7)	4.2 (3.7, 4.7)
*p*-Value	0.869	0.185	0.211	0.176

HbA1c (mmol/mol)				

<48	37.0 (34.5, 39.3)	8.0 (6.8, 9.0)	2.6 (2.0, 3.3)	4.3 (3.7, 4.8)
>48	35.6 (32.7, 38.4)	5.5 (4.8, 6.3)	3.0 (2.4, 3.6)	3.7 (3.0, 4.3)
*p*-Value	0.476	**<0.001**	0.510	0.182

HRQoL by proxy (score)				

<70	36.0 (33.0, 38.9)	6.2 (5.0, 7.4)	3.0 (2.3, 3.7)	3.4 (2.6, 4.1)
>70	36.4 (34.0, 38.9)	7.2 (6.2, 8.2)	2.7 (2.1, 3.4)	4.4 (3.9, 4.8)
*p*-Value	0.821	0.183	0.579	**0.016**

HRQoL child				

<70	32.9 (29.5, 36.0)	7.0 (5.6, 8.2)	2.8 (2.1, 3.5)	3.3 (2.6, 4.0)
>70	38.4 (36.6, 40.3)	6.6 (5.7, 7.6)	2.9 (2.3, 3.4)	4.4 (3.9, 4.9)
*p*-Value	**0.003**	0.621	0.849	**0.009**

Education parents				

Mother lower	35.6 (32.3, 38.9)	6.3 (5.1, 7.6)	2.8 (2.0, 3.6)	3.8 (3.0, 4.6)
Mother higher	36.8 (34.2, 39.1)	7.3 (6.2, 8.4)	3.0 (2.4, 3.5)	4.1 (3.5, 4.7)
*p*-Value	0.568	0.259	0.653	0.615
Father lower	36.0 (33.0, 39.3)	6.7 (5.3, 8.1)	2.9 (2.2, 3.5)	3.9 (3.2, 4.7)
Father higher	36.9 (34.6, 39.2)	6.9 (5.8, 8.0)	2.8 (2.2, 3.5)	3.9 (3.3, 4.6)
*p*-Value	0.335	0.801	0.934	0.968

Economic group				

Better than average	37.1 (34.3, 39.9)	6.9 (5.6, 8.1)	3.1 (2.5, 3.7)	3.8 (3.1, 4.5)
Average	35.4 (33.0, 37.8)	6.8 (5.7, 7.8)	2.8 (2.2, 3.5)	3.9 (3.4, 4.5)
Worse than average	36.0 (−78.4, 159.3)	7.5 (−11.6, 26.6)	2.5 (−3.8, 8.8)	2.5 (−3.8, 8.8)
*p*-Value	0.665	0.934	0.737	0.416

Family situation				

Parents living together	36.7 (34.5, 39.2)	6.5 (5.6, 7.5)	3.1 (2.5, 3.6)	4.0 (3.4, 4.6)
Shared custody/single	35.1 (31.4, 38.5)	7.4 (5.9, 9.0)	2.6 (1.8, 3.5)	3.6 (2.9, 4.4)
*p*-Value	0.444	0.301	0.398	0.437

*Note:* One way ANOVA or *T*-test. Data in bold are statistically significant.

^a^DTSQ-Parent = Item 1, 5–9, 11−12.

^b^Perceived diabetes control Item 2, 3 (How well controlled do you feel your child's diabetes has been lately? How often have you felt that your child's blood sugars have been too high lately?).

^c^Item 4 = How often have you felt that your blood sugars have been too low lately?

^d^Item 10 = How bothered are you by any discomfort or pain caused by your current one treatment?

**Table 5:** The association between treatment satisfaction and background and clinical factors in children with Type 1 diabetes aged 8–12 years, analyzed with linear regression.

Background and clinical factors	*B*	*β*	95% CI^σ^	*p*-Value
Constant	27.83	—	10.20, 45.46	0.003
Child's age	−0.03	−0.08	−1.15, 1.09	0.953
Sex^a^	1.37	0.11	−1.73, 4.46	0.379
Child's HRQoL^b^	0.15	0.42	0.06, 0.25	**0.002**
Diabetes duration	0.12	0.01	−0.52, 0.54	0.965
HbA1c	−0.09	−0.20	−0.22, 0.03	0.127
*R* ^2^	0.29	—	—	**0.005**

*Note:* Dependent variables: Treatment satisfaction (Item:1, 5–9, 11–12). Data in bold are statistically significant.

Abbreviations: CI, confidence interval; HRQoL, health-related quality of life.

^a^Girls = 1, boys = 2.

^b^Health related quality of life.

^σ^Confidence interval.

## Data Availability

The data that support the findings of this study are available from the corresponding author upon reasonable request.
